# Median artery persistence in coexistence with a bifid median nerve and interconnections of the median with the ulnar nerve and vice versa

**DOI:** 10.1007/s00276-023-03183-y

**Published:** 2023-06-27

**Authors:** Maria Piagkou, George Tsakotos, George Triantafyllou, Christos Koutserimpas, Trifon Totlis, Vasilios Karampelias, Konstantinos Natsis

**Affiliations:** 1 Department of Anatomy, Faculty of Health Sciences, School of Medicine, National and Kapodistrian University of Athens, 75 Mikras Asias Str., Goudi, 11527 Athens, Greece; 2 Department of Orthopaedics and Traumatology, “251” Hellenic Air Force General Hospital of Athens, Athens, Greece; 3 Department of Anatomy and Surgical Anatomy, Faculty of Health Sciences, School of Medicine, Aristotle University of Thessaloniki, Thessaloniki, Greece

**Keywords:** Persistent median artery, Bifid, Median nerve, Ulnar nerve, Martin–Gruber anastomosis, Marinacci anastomosis

## Abstract

**Purpose:**

This report describes a bilateral persistent median artery (PMA) originating from the ulnar artery and terminating at different levels of the upper limb. The PMA coexisted with a bilateral bifid median nerve (MN) and two bilateral interconnections (ICs characterized with the symbol -) of the MN with the ulnar nerve (UN) (MN-UN) and a unilateral reverse IC (UN-MN). Emphasis was given to the artery’s developmental background.

**Methods:**

The PMA was identified in an 80-year-old formalin-embalmed donated male cadaver.

**Results:**

The right-sided PMA terminated at the wrist, posterior to the palmar aponeurosis. Two neural ICs were identified: the UN joined the MN deep branch (UN-MN), at the forearm’s upper third, and the MN deep stem joined the UN palmar branch (MN-UN), at the lower third (9.7 cm distally to the 1st IC). The left-sided PMA ended in the palm giving off the 3rd and 4th proper palmar digital arteries. An incomplete superficial palmar arch was identified by the contribution of the PMA, radial, and ulnar arteries. After the MN bifurcation into superficial and deep branches, the deep branches formed a loop, that was penetrated by the PMA. The MN deep branch communicated with the UN palmar branch (MN-UN).

**Conclusions:**

The PMA should be evaluated as a causative factor of carpal tunnel syndrome. The modified Allen's test and the Doppler ultrasound may detect the arterial flow and the angiography may depict the vessel thrombosis in complex cases. PMA could also be a "salvage" vessel for the hand supply, in radial and ulnar artery trauma.

## Introduction

The radial and ulnar arteries (RA and UA) are the primary forearm’s blood suppliers. During the first gestational trimester, the median artery typically supplies the entire hand, and after the 8^th^ gestational week, it regresses to a small vessel accompanying the median nerve (MN) [17]. Rodríguez-Niedenführ et al. [11, 13], proposed the theory of the selective enlargement or regression of a dispersed capillary plexus of the dorsal aorta that enters the limb bud and ramifies into capillaries, and not by budding from a main axial trunk. The persistent median artery (PMA) as regressing to the wrist area is considered a typical small nutrient vessel to the MN proximal segment, contrariwise to the PMA enlargement and extension in the hand, that is considered as a variant [20]. The PMA variant morphology (long or short vessel) is characterized, based on the area of its termination (1) hand area-palmar type, the variant and (2) wrist area-antebrachial type, the typical form). Antebrachial PMA has a higher prevalence (34%) compared to the variant form of the PMA, the palmar type (8.6%) [18]. Thus, the PMA should be defined as the median artery (of a "large" diameter) identified after birth and contributing to the hand supply [11]. The PMA in the carpal tunnel (CT) may coexist with the other neurovascular variants, with the more common being the MN bifurcation and the incomplete formation or absence of a superficial palmar arch (SPA) [10, 19]. The current report describes in detail the PMA typical and variant morphology, taking into consideration the artery’s origin, course, and area of termination, as well as the coexisting neural variants, emphasizing their laterality. Furthermore, the clinical implications of these variants are also discussed.

## Case description

An 80-year-old donated male cadaver was bilaterally dissected at the upper limb. The body was donated to the Anatomy Department, through the “Anatomical Gift Program” after written informed consent. In both upper limbs, the PMA originated from the UA and coexisted with a bifid MN and an interconnection (IC) of the MN deep branch with the ulnar nerve (UN) palmar branch (Martin–Gruber IC), at the forearm upper and lower thirds. Unilaterally (right side), an IC of the UN palmar branch with the MN deep branch was identified (Marinacci IC), at the forearm upper third.

**Fig. 1:**
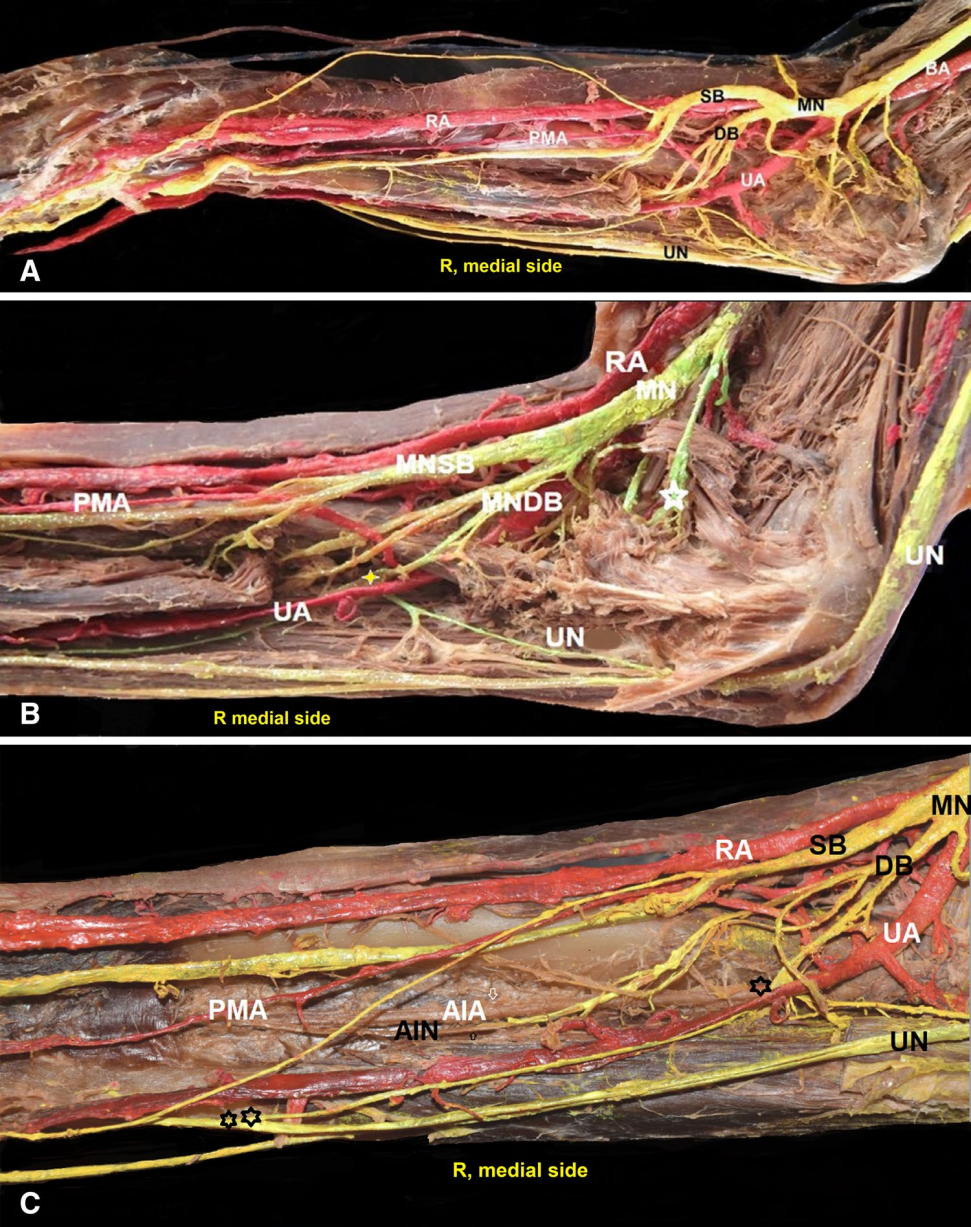
**A-C** The right-sided (R) persistent median artery (PMA) of antebrachial type and origin from the ulnar artery (UA). The median nerve (MN) bifurcation into superficial and deep branch (SB and DB) and the PMA proximal ascending course, **B** thin asterisk—the interconnection (IC) ulnar nerve (UN) and the MN deep branch, at the forearm upper third, thick asterisk-muscular branch to pronator teres muscle, **C** one asterisk—the IC (UN with the MN deep branch), at the forearm upper third, two asterisks—the IC (MN deep stem with the UN palmar branch), 9.7 cm distally to the IC (UN with the MNDB), RA—radial artery, AIA—anterior interosseous artery (white arrow), and AIN—anterior interosseous nerve (black arrow)

**Fig. 2:**
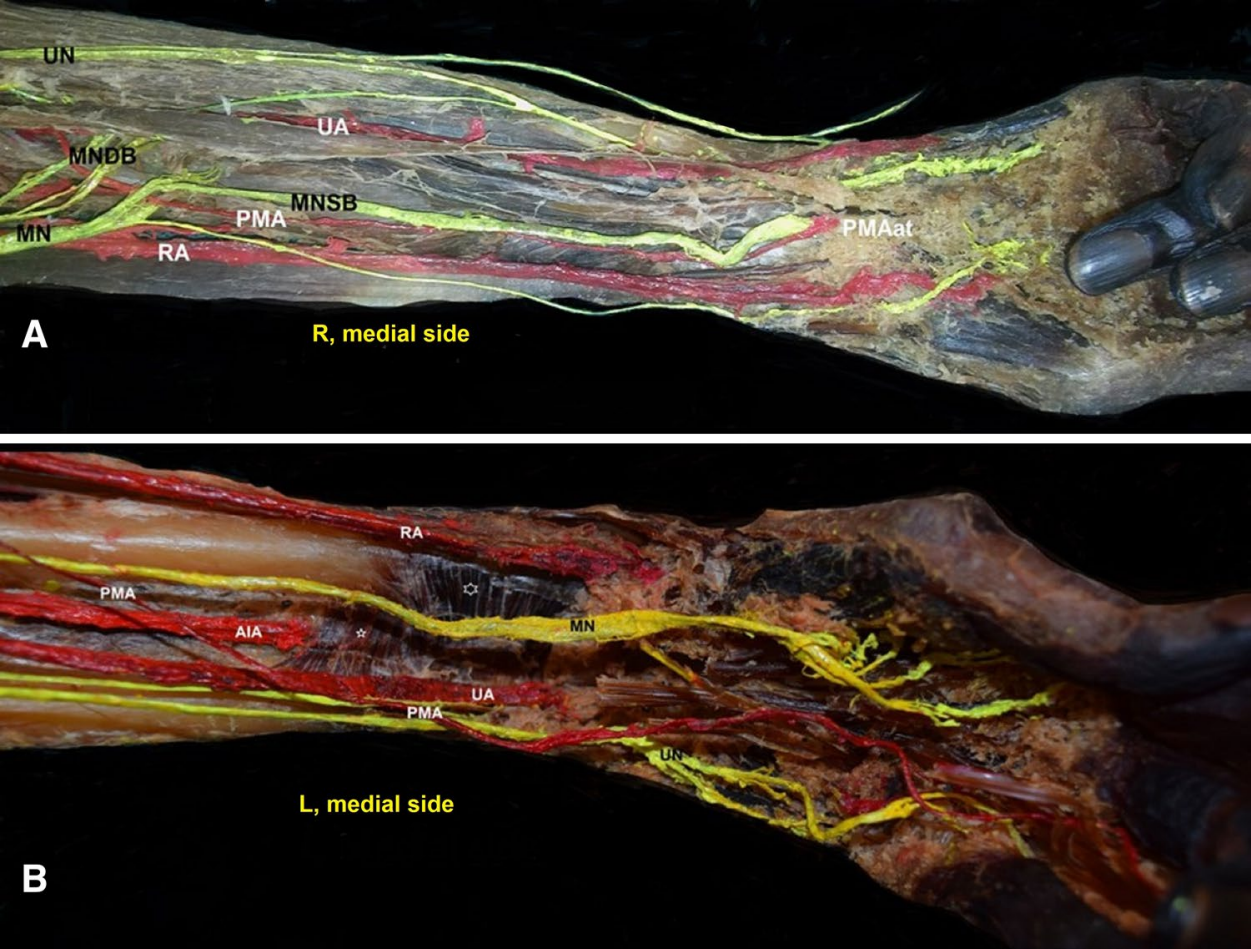
**A** Right (R) and **B** left (L) side specimens of the persistent median artery (PMA) of antebrachial type (PMAat) and palmar type. **Α** The PMAat anterior position and the median nerve superficial branch (MNSB), **B** PMA of palmar type, AIA—anterior interosseous artery penetrating the deep muscle surface of the quadratus pronator muscle (small asterisk), big asterisk—the superficial muscle surface of the pronator quadratus, RA—radial artery, and UA—ulnar artery

On the right side, the antebrachial PMA emanating posterior to the pronator teres muscle reached an anterior position in relation to the MN superficial branch (Fig. 1), and terminated at the wrist, posterior to the palmar aponeurosis (level of the flexor digitorum superficialis tendon) (Fig. 2). The MN is divided into superficial and deep stems (pronator teres muscle level). The superficial stem gave off a thin branch to the palm, and a thick branch that further bifurcated. The PMA passed between these branches’ divisions. A muscular branch to the flexor digitorum superficialis was given off from the superficial stem, with posterior course to the PMA and through the transverse carpal ligament. In the forearm upper third, the MN deep stem supplied the pronator teres, the flexor carpi radialis, and the palmaris longus muscles. After the IC of the UN with the MN deep stem (1st IC-Marinnaci), the MN deep stem gave off a muscular branch to the flexor carpi ulnaris muscle. In the forearm lower third, a branch of the MN deep stem interconnected with the UN palmar branch (2nd IC, Martin–Gruber) (Fig. 1).

**Fig. 3:**
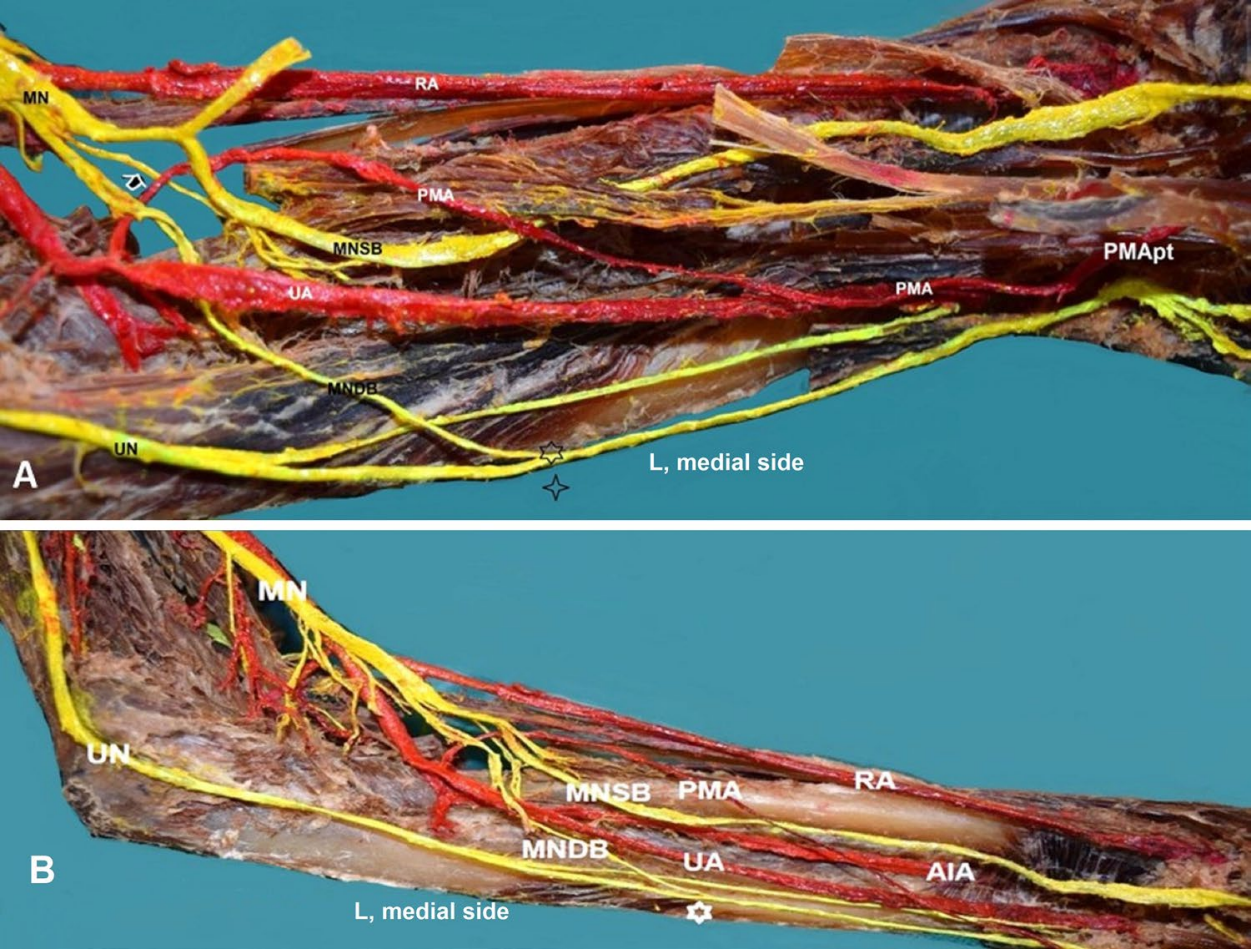
**A** Left-sided (L) persistent median artery (PMA) of palmar type (PMApt) originated from the ulnar artery (UA). The loop (arrow) created from the MN deep branches (MNDB). The PMA crossing with the MN superficial branch (MNSB), black asterisks—the MNDB interconnection with the ulnar nerve (UN) palmar branch, **B** white asterisk—MNDB interconnection with the UN palmar branch, AIA—anterior interosseous artery (from the UA) posterior course in relation to the MNSB, and RA—radial artery

On the left side, the palmar PMA, originating 3.5 cm distally to the brachial artery bifurcation, proximal to its origin from the UA, followed an ascending course lateral and proximal to the MN division into superficial and deep branches and after crossing the MN superficial branch, descended to reach an anteromedial position at the wrist, medial to the ulnar nerve (Fig. 3). The PMA continued medially to the flexor digitorum superficialis and terminated at the palm, giving off the 3rd and 4th proper palmar digital arteries. An incomplete SPA was identified (Fig. 2). The MN, after its medial course concerning the brachial artery supplied the pronator teres muscle and 2 cm distally, divided into a superficial and a deep branch. The MN superficial branch supplied the flexor digitorum superficialis and thereafter passed between flexors digitorum profundus and superficialis, posterior to PMA and transverse carpal ligament, distributing to the thumb, index, and middle finger. The MN deep branch was further divided into medial and lateral branches, that joined into a loop, through which coursed the PMA. The MN deep medial branches supplied the flexor digitorum profundus and gave off an IC to the UN palmar branch (Martin–Gruber IC). The UN following a typical origin and course medial to the brachial artery and between the flexor carpi ulnaris two heads was divided into a palmar and a dorsal branch, 3.4 cm distally to the medial epicondyle. The UN palmar branch after communication with the MN deep branch is distributed to the medial third of the palm. The UN dorsal branch coursed below the flexor carpi ulnaris tendon and terminated in the dorsal surface of the palm. The MN deep lateral branches are distributed to the flexor pollicis longus muscle (Fig. 3).

## Discussion

The current case highlights the coexistence of a bilateral PMA of origin from the UA, and variant termination (at the hand and wrist area) with a bilateral (MN-UN) and a unilateral (UN-MN) IC. Both PMAs had an anteromedial position concerning the MN, contrariwise to Haladaj et al.’s study [2], in which only the 20% of the PMA cases had an anteromedial position and the rest 40% had an anterolateral and anterior position in relation to the MN (per each). Solewski et al. [18] identified the PMA highest prevalence (of 30.2%) in fetuses, followed by neonates and infants (18.6%) and adults (7.5%). The PMA higher prevalence (9.9%) was identified in cadaveric studies, followed by ultrasonographic (9.7%) and radiological (8%) studies [18]. Developmentally, the axillary, brachial, and interosseous arteries are derived from a main axial trunk, which extends from the axilla to the fingers. During early embryonic life, the median and the interosseous arteries represent the main hand suppliers. As development proceeds, some vessels enlarge and assume their form, while others regress. PMA regression starts after the 8^th^ gestational week and yields to the UA and RA. Postnatally, the MA may persist and originate from the anterior interosseous artery or directly from the UA [17].

In the current report, the PMA originated from the UA, bilaterally. Saenz et al. [15] in their cadaveric study identified a variable PMA origin, from the UA in 48%, from the anterior interosseous artery in 36%, and the common interosseous artery in 16% [15]. Recent cadaveric reports pointed out the coexistence of PMA with superficially located vessels, absent vessels, and ectopic vessels in the upper limb [4, 15]. Kalinowski et al. [4] pointed out the coexistence of ectopic origins of PMA and common interosseous artery from the RA and their coexistence with a superficial brachial artery. In the current report, the left-sided PMA contributed to an incomplete SPA. Zarzecki et al. [19] found the prevalence of PMA coexistence with an incomplete SPA at 11.9%. This coexistence may have clinical consequences, as the PMA following the route to join the SPA often accompanies in proximity the MN, resulting in several syndromes, such as the carpal tunnel syndrome (CTS), the anterior interosseous nerve syndrome, and the pronator teres syndrome, increasing the degree of hand ischemia [19]. Thus, the hand collateral supply is more likely to be compromised by the PMA intraoperative ligation. The PMA may also coexist with muscles’ atypia, like a reverse or absent palmaris longus [8, 16], an accessory lumbrical [1], and an accessory flexor carpi ulnaris muscle [16]. In the current case, no atypical muscle was identified on both sides.

In the current case, both PMAs of origin from the UA divided the MN in the forearm, as Saenz et al. [15] recorded in 100% of their sample. In the current report, a bilateral IC of the MN deep branch with the UN palmar branch coexisted with a unilateral IC of the UN with the MN deep stem. The PMA may coexist with brachial plexus variants, such as the bifid MN [10, 16] and the IC (MN-UN) [6]. Such ICs are classified based on their location, fibers’ type (sensory, motor, or both), and direction (from MN to UN, or vice versa from UN to MN), and are of paramount importance as may skew electrodiagnostic findings, possibly resulting in misdiagnosis and iatrogenic injury [6, 14]. Maya-Gonzalez et al. [7] described a rare bilateral (MN-UN) and (UN-MN) IC, associated with injury of the MN and UN in the CT and Guyon's canal, emphasizing the difficulty of these variants’ identification in their asymptomatic form, as well as their identification and interpretation (based on the motor or sensory deficit) when nerve injury occurs. Rodriguez-Niedenfurh et al. [12] identified the IC (MN-UN) in 22.9% (being unilateral in 81.3% and bilateral in 18.7%). They identified the IC originating from the MN superficial branch (in 47.3%), from the anterior interosseous nerve (in 31.6%), and from the MN (in 10.6%) [12]. Although the IC (UN-MN) has not been previously studied on cadavers, its prevalence has been estimated in 0.7% for the classic type of IC, based on electro-neuro-myographic studies [15]. It should be noted that MN originates from the brachial plexus (C5-T1 roots), after the medial and lateral cords’ fusion, and the UN originates from the brachial plexus medial cord (C8-T1). Hence, there is some common origin of these two main forearm nerves. Thorough clinical examination if of paramount importance for the diagnosis of the numerous entrapment syndromes of the MN and UN in the forearm, including the pronator teres syndrome, the anterior interosseous nerve syndrome, and the CTS (regarding the MN), as well as the cubital tunnel syndrome and the ulnar tunnel syndrome (regarding the UN) [6].

Surgeons utilizing the forearm palmar approaches, such as the Henry approach, for the internal fixation of radius and/or ulnar fractures and or proximal and/or distal radio-ulnar joint reduction and stabilization should have in mind this area’s variants. Meticulous exploration is of utmost importance to avoid injury [3]. Furthermore, numerous forearm procedures may be complicated by the presence of these variations, including forearm flaps, harvesting of the palmaris longus or the flexor carpi radialis for the treatment of basal thumb arthritis, as well as angioplasties. The exploration of the carpal tunnel and Guyon canal should also be performed with caution. CTS is a common orthopaedic issue, treated mainly surgically with release of the MN. This routine procedure may be complicated by the possible variants in the area, as their total prevalence in patients suffering from CTS is 63.5% (PMA, bifid MN, a transverse carpal muscle, and a subligamentous recurrent branch of the MN) [10].

The CT walls are inflexible; hence, any thickening of the components may compress the MN and its branches. A PMA when increased to 3 mm diameter could lead to CTS, in paresthesia and pain in the area innervated by the MN [10, 18, 19]. There is no evidence, suggesting that a PMA may result in CTS, per se. A PMA could be an independent risk factor for CTS, in the presence of pathology, such as the presence of an internal thrombus, of an aneurysm or a calcified plaque formation [9]. The occurrence of a thrombosed PMA or any other pathology involving the artery that perforates the MN can indirectly injure the MN. Direct, mechanical injury can be caused by the PMA dilatation with MN [9].

In addition, arteriovenous malformations may have arisen from pre-existing vessels, as Krisnamurthy et al. [5] identified. They were referred to a high flow arteriovenous malformation fed by a PMA. Although the PMA may constitute a significant supply to the hand, and to the MN [18] may also compress on the MN [15] and affect its conduction velocity [15]. Thus, paresthesia, pain in the forearm or at the wrist and symptoms mimicking CTS (usually acute) may occur [9].

In CT, the bifid MN may confuse during a procedure or may cause intraoperative nerve injury [10]. The PMA is associated with a bifid MN [10] and an incomplete SPA [15]. The knowledge of the SPA variant formation is essential, especially in complex hand surgery, and thus, angiographic investigation may be further useful [7]. The presence of ICs between MN and UN and vice versa may further complicate the CTS surgical release, because the functional loss caused by the MN entrapment differs in the presence of an IC. Additionally, those ICs may even explain some cases of injuries in the forearm that are not reflected in the hand muscles.

### Report’s limitations

A limitation of the current report is referred to the quality of the hand tissues, mainly regarding the poor elasticity, that affected dissection and final specimen quality. Thus, photographic documentation regarding PMA branches' distribution in hand was not provided. In addition, cutaneous from muscular branches could not be identified immunohistochemically (identification of the type of fibers, i.e., motor, or sensory), although the dissection was careful, and the UN branching pattern was partially identified to supply the skin (terminal cutaneous branches).

## Conclusion

The symmetrical or asymmetrical combination of variants in the wrist area described in the current report has clinical significance. The bilateral PMA coexisted with a bilateral bifid MN, a bilateral Martin–Gruber interconnection, a unilateral incomplete SPA, and a unilateral Marinacci interconnection. The asymmetrical (of different termination levels) bilateral existence of PMA is quite rare and is often associated with neural variants that may further complicate wrist surgery. It is also of note that the PMA is the main nutrient artery of the MN, and therefore, it should be preserved. Surgeons should be aware of all these variants and assess the feasibility of safe surgery through preoperative examination.

## Data Availability

Data will be available with the corresponding author for further reference.
